# Super-Enhancer Drives *THBS3* Expression to Regulate the Proliferation and Differentiation of Bovine Muscle Stem Cells

**DOI:** 10.3390/ani15172615

**Published:** 2025-09-06

**Authors:** Han Huang, Yongwang Zhang, Kehe Cen, Chaoxia Zou, Leyi Wang, Jiaqi Lu, Haiming Mai, Jinquan Ding, Junbo Pan, Zeyang Zhao, Junming Li, Yanfei Deng, Jingwei Wei, Deshun Shi, Yingming Wei, Ruimen Zhang

**Affiliations:** Guangxi Key Laboratory of Animal Breeding, Disease Control and Prevention, Guangxi University, Nanning 530004, China; 2318301013@st.gxu.edu.cn (H.H.); zywwang160521@163.com (Y.Z.); 2218391002@st.gxu.edu.cn (K.C.); m15240667014@163.com (C.Z.); wangleyi959@163.com (L.W.); 18771164460@163.com (J.L.); 13517795161@163.com (H.M.); m18934719363@163.com (J.D.); y1327785625@163.com (J.P.); 13377069520@163.com (Z.Z.); y1056196297@163.com (J.L.); yanfei-dun@163.com (Y.D.); weijw99@sina.com (J.W.); ardsshi@gxu.edu.cn (D.S.)

**Keywords:** bovine, *THBS3* gene, super-enhancer, myogenesis, MuSCs

## Abstract

Skeletal muscle, the primary meat-producing tissue in bovines, is regulated by a complex transcriptional network during development. The role of *Thrombospondin 3* (*THBS3*) and its associated super-enhancer (SE) in this process remains largely unknown. Here, by integrating multi-omics data, we identified *THBS3* as a novel core regulator of myogenesis, orchestrated by a cognate super-enhancer (*THBS3*-SE). Functional assays demonstrated that *THBS3* knockdown significantly promoted the proliferation and myogenic differentiation of bovine muscle stem cells (MuSCs) and accelerated their commitment to a fast-twitch fiber fate. Transcriptomic analysis linked *THBS3* function to key signaling pathways controlling muscle growth, especially the mechanistic target of rapamycin (mTOR) signaling pathway. Mechanistically, we found that distal enhancers within the *THBS3*-SE loop to the *THBS3* promoter drive its transcription, and CRISPR-based interference of these enhancers recapitulated the pro-myogenic effects of *THBS3* knockdown. Collectively, our findings unveiled a *THBS3*-SE-mediated regulatory axis that critically governed bovine MuSCs’ fate. Targeting this axis may offer a novel strategy for improving beef production efficiency.

## Simple Summary

Beef cattle are a major source of animal protein worldwide. Understanding how muscle tissue forms and develops can lead to improvements in both the quantity and quality of beef. In this study, we combined multiple types of biological data to investigate a key gene called *THBS3*, which plays a significant role in muscle development in cattle. We found that *THBS3* is activated by a neighboring regulatory region known as a super-enhancer (*THBS3*-SE). This region physically interacted with the *THBS3* gene to influence the formation of skeletal muscle. Our results revealed an important regulatory pathway that controlled muscle formation in cattle, offering new insights and potential targets for breeding strategies aimed at enhancing beef production.

## 1. Introduction

Skeletal muscle represents a critical determinant of meat production in cattle, where developmental processes (myogenesis) directly influence yield and quality. Myogenesis initiates with the activation, proliferation, and subsequent myogenic differentiation of muscle stem cells (MuSCs). Following differentiation, MuSCs fuse into multinucleated myofibers, constituting the fundamental structural units of skeletal muscle [1]. Consequently, the ultimate quality of skeletal muscle (encompassing myofiber number and size) is fundamentally determined by the proliferative capacity and myogenic differentiation efficiency of MuSCs.

Myogenesis is orchestrated by a multi-tiered molecular network that ensures precise differentiation [2]. Core myogenic regulators, including *PAX3/7*, act in a stage-specific manner to govern MuSCs proliferation and inhibit differentiation, thereby maintaining the stem cell pool [3]. Myogenic regulatory factors (MRFs) drive MuSCs differentiation [4,5], while Myocyte enhancer factor 2 (*MEF2*) coordinates the transcription of myogenic genes [6], collectively forming the core regulatory network. Epigenetic elements, particularly super-enhancers (SEs), critically specify myocyte identity and temporally coordinate differentiation gene expression [7]. SEs comprise large genomic regions (typically 8–20 kb) of densely clustered enhancer elements enriched in active histone modifications (e.g., H3K27ac, H3K4me1), enabling robust, cell-type-specific transcription of target genes [8,9]. Studies demonstrated that SEs interacted with target gene promoters via higher-order chromatin structures (e.g., chromatin loops), serving as pivotal drivers of muscle-specific gene expression [10]. For instance, *MyoD1* and *CASZ1* co-occupy specific SEs to facilitate chromatin looping, thereby activating muscle differentiation gene networks [11]. Notably, SEs can even direct fibroblast reprogramming toward myogenic lineages, underscoring their potent regulatory capacity [12,13]. However, despite their established role in model organisms, the identity and functional relevance of SEs in agriculturally important species such as bovines remained largely unexplored. The integration of multi-omics approaches represents a powerful strategy to bridge this gap, enabling genome-wide annotation of SEs and functional validation of their roles in key traits such as skeletal muscle development.

*Thrombospondin-3* (*THBS3*), an adhesion glycoprotein, modulates diverse biological processes including oncogenesis, cardioprotection, cutaneous repair, and skeletal maturation [14,15,16,17]. Gradually, there have been reports in the literature that *THBS3* functions in muscle biology. Zhao et al. reported correlations between *THBS3* expression and muscle drip loss in swine, implying a potential influence on meat quality [18]. Intriguingly, *THBS4* (a *THBS3* paralog) promoted myofibrillogenesis and skeletal muscle regeneration in murine models, significantly ameliorating atrophy [19]. Our prior transcriptomic (RNA-seq) analysis of bovine MuSCs across proliferation (GM) and differentiation (DM) stages revealed *THBS3* upregulation during DM, indicating its potential involvement in the proliferation and differentiation of bovine MuSCs. By integrating these data with H3K27ac ChIP-seq profiles, we identified a novel SE associated with *THBS3* in bovine MuSCs, which we designated *THBS3*-SE. Nevertheless, whether *THBS3*-SE directly controls *THBS3* expression and participates in bovine myogenesis remains unknown.

Therefore, we hypothesized that the *THBS3*-SE is a critical upstream regulator of bovine myogenesis, directly modulating *THBS3* expression to influence muscle development. To test this, we aimed to elucidate the functional impact of *THBS3* on bovine MuSCs through molecular intervention strategies and to preliminarily identify the molecular mechanism by which *THBS3*-SE drives *THBS3* regulation of muscle generation. Our research not only enriches the regulatory network governing bovine muscle development but also provides valuable targets for molecular breeding in beef cattle.

## 2. Materials and Methods

### 2.1. Multi-Omics Joint Analysis

All omics analyses were conducted using bovine MuSCs isolated from the longissimus dorsi muscle tissue of fetal cattle, based on sequencing results previously analyzed by Zhang et al. at Guangxi University. ChIP-seq and RNA-seq analyses were performed during the GM and DM stages of MuSCs. ChIP-seq was performed using an H3K27ac-specific antibody, and the ROSE algorithm was employed to identify SE. The signal intensity (0–100) within each constituent enhancer was calculated using the H3K27ac BAM file, and the constituent enhancers were ranked based on their H3K27ac signal intensity to obtain the SEs. RNA-seq analysis identified gene sets differentially expressed between GM and DM. To identify differentially expressed genes (DEGs) between the two groups, Differential analysis of the obtained clean data was performed by DESeq2 software (DESeq2 package version: 1.48.1), with a screening criterion of log_2_ (Fold Change) > 1.5 and *p*-values < 0.05.

Genes located within 1 Mb of SEs in the genome are termed SE-regulated genes [20]. The SE-regulated gene set from ChIP-seq was correlated with differentially expressed genes from RNA-seq to obtain the number, distribution, and expression levels of differentially expressed SE-regulated genes. At the same time, CLUEGO was used to perform GO and KEGG analysis of SE-regulated genes to predict the biological processes most relevant to SEs. All data is hosted at Guangxi University and is freely available by contacting the corresponding author.

### 2.2. Sample Preparation

Tissue samples from the heart, liver, spleen, lungs, kidneys, small intestine, stomach, front legs, hind legs, longest back muscle, and brain of embryonic-stage cattle (90 days) and the longest back muscle tissue of adult cattle (18 months) were stored at −80 °C. Bovine MuSCs and 293T cells were stored in liquid nitrogen. The above samples were consistent with those reported by Zhang et al. [21].

### 2.3. Cell Culture

Refer to the experimental methods of previous studies [22]. Culture primary bovine MuSCs and complete the identification process (Supplementary Figure S1). MuSCs were cultured in GM supplemented with 10–20% FBS (Hyclone, Logan, UT, USA) and antibiotics (1% penicillin and streptomycin) at 37 °C in a 5% CO_2_ environment. When cell confluence reached 90–100%, the medium was switched to DM composed of 98% DMEM (Gibco, Waltham, MA, USA) and 2% horse serum to induce myogenic differentiation of MuSCs.

### 2.4. Total RNA Extraction

TRizol reagent (Invitrogen, Carlsbad, CA, USA) was used to extract total RNA from the cell and tissue samples in accordance with the instructions of the manufacturer.

### 2.5. Quantitative Reverse Transcription Polymerase Chain Reaction (RT-qPCR)

The total RNA extracted from cells and tissues was reverse transcribed into cDNA using reverse transcriptase (ABclonal, RK20408, Wuhan, China), and qPCR was performed using SYBR Green reagent (ABclonal, RK21219, Wuhan, China) according to the manufacturer’s instructions. β-actin was used as an internal reference, and the 2^−ΔΔCt^ method was used for calculation. Each group contains at least three biological replicates. Supplementary Table S1 lists all primer sequences used.

### 2.6. Synthesis and Transfection of Small Interfering RNA (siRNA)

Based on the *THBS3* transcript sequence, RiboBio designed interference sequences and synthesized three si-*THBS3* (RiboBio, Guangzhou, China). The negative control was also purchased from RiboBio. Supplementary Table S2 lists the designed interference sequences. Transfection was performed according to the instructions for riboFECT mRNA Transfection Reagent (RiboBio, C11055-1, Guangzhou, China).

### 2.7. Cell Counting Kit-8 (CCK-8) Assay

Cell proliferation was assessed using CCK-8 (Vazyme, Nanjing, China) according to the manufacturer’s instructions. There were six independent replicates for each treatment group.

### 2.8. 5-Ethynyl-20-Deoxyuridine (EdU) Assay

Proliferation of MuSCs was assessed using the BeyoClick™ EdU Cell Proliferation Kit (Beyotime, C0071S, Shanghai, China) with Alexa Fluor 555. Three independent replicates were performed for each treatment group. We used ImageJ 1.8.0 for cell counting. A uniform fluorescence intensity threshold was set for each field of view, and cells above this threshold were classified as EdU-positive cells. The formula for calculating the EdU-positive cell rate (%) for each field of view is (number of EdU-positive cells/total number of cells) × 100%.

### 2.9. Western Blot (WB)

Our experimental procedures were based on previous experimental reports [22]. All primary antibodies were from ABclonal (Wuhan, China). After being electrophoresed and transferred, the primary antibodies (MyoD1, 1:1000; CDK2, 1:1000; PCNA, 1:1000; MyHC, 1:1000; MyH2, 1:1000; MyH4, 1:1000; β-actin, 1:5000) were used to bind target protein overnight at 4 °C. The membranes were then washed with TBS-Tween and incubated with the corresponding secondary antibody, goat anti-rabbit IgG (ABclonal, Wuhan, China), at room temperature for 1 h. Protein bands were detected using Super Signal West Femto reagent purchased from Thermo (Thermo Scientific, Waltham, MA, USA). Images were analyzed using ImageLab (Bio-Rad, Hercules, CA, USA). Each group contains at least three biological replicates.

### 2.10. Immunofluorescence

Myoblasts were incubated overnight at 4 °C with primary antibodies against MyHC (ABclonal, Wuhan, China) and Pax7 (ABclonal, Wuhan, China). The cells were then washed three times with PBS and incubated at room temperature with 5% bovine serum albumin (BSA) at room temperature for 1.5 h in the dark. DNA was stained with 10 μg/mL DAPI (4′,6-diamidino-2-phenylindole, cell signaling) and observed under an EVOS fluorescence microscope (Thermo, Waltham, MA, USA) after washing the cells three times with PBS. Each group contains at least three biological replicates.

### 2.11. RNA-Seq Analysis

The total RNA of bovine MuSCs that had been induced to differentiate for 4 days (*n* = 4) in the control group (si-NC) and the treatment group (si-*THBS3*) was extracted using the TRizol kit and transferred to Gene Denovo (Guangzhou, China) for RNA sequencing. To identify differentially expressed genes (DEGs) between the two groups, differential analysis of the obtained clean data was performed by DESeq2 software (DESeq2 package version: 1.48.1) using the following screening criteria: log_2_ (Fold Change) > 1.5 and *p*-values (*p* adj) < 0.05. The identified DEGs were then subjected to gene ontology (GO) and Kyoto Encyclopedia of Genes and Genomes (KEGG) pathway enrichment analysis. All data is hosted at Guangxi University and is freely available by contacting the corresponding author.

### 2.12. Chromatin Conformation Capture (3C)

The 3C protocol has been previously described by Jessica et al. [23]. HindIII restriction sites were designed to detect possible interaction sites between *THBS3*-SE and the *THBS3* promoter. The ligation products were quantified by RT-qPCR. The primer sequences used are shown in Supplementary Table S2.

### 2.13. Dual-Luciferase Reporter System Assay

*THBS3*-SE was composed of 16 enhancer elements in bovine genomes. Enhancers E1 to E16 were ligated into the PGL3-Promoter vector and then transfected into 293T cells for activity verification. Luciferase activity was subsequently measured using the Luc-Pair TM Duo-Luciferase HS Assay Kit (GeneCopoeia, LF004, Rockville, MD, USA) following the manufacturer’s protocol. The Enhancer primer sequences used are shown in Supplementary Table S1.

### 2.14. Vector Construction and Transfection

Px330a dCas9-E15 and Px330a dCas9-E16 inhibitory vectors were designed and synthesized by GenScript (Kingsley Biologicals, Stanford, NJ, USA), and the detailed vector sequences are shown in Supplementary Table S2. Experiments were performed according to the instructions of LipofectamineTM 3000Reagent (Thermo Fisher, L3000008, Waltham, MA, USA). Transfection complexes of plasmid vectors were configured and then transfected into MuSCs.

### 2.15. Enhancers E15 and E16 Combined Motif

The binding motifs for E15 and E16 were generated using the FIMO module of the MEME suite. Transcription factors were selected from the HOCOMOCO V12 database. The significance threshold of *Q*-value < 0.05 was applied to identify transcription factors with predicted binding sites within these two elements. The relevant material information has been uploaded to Supplementary Files.

### 2.16. Statistical Analysis

All results were expressed as the mean ± standard errors of the means (SEMs) of the experiments. Multiple group comparisons were analyzed by one-way ANOVA using Graphpad Prism version 9.0 (Graphpad Software, La Jolla, CA, USA), while comparisons between two groups were analyzed using *t*-tests. Differences were considered significant when *p* ≤ 0.05 was obtained.

## 3. Results

### 3.1. Analysis of Genes Regulated by Bovine Muscle Super-Enhancer

**Figure 1:**
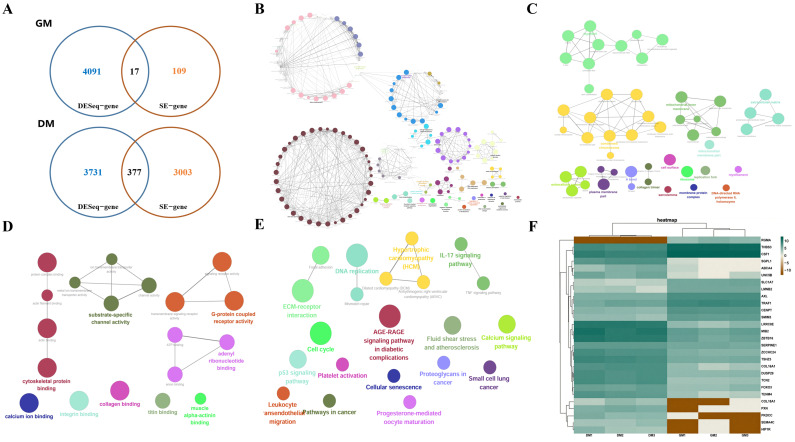
Analysis of genes regulated by bovine muscle super-enhancer. (**A**) Number of SE target genes differentially expressed in bovine MuSCs during the GM and DM. (**B**) GO biological process clustering heat map. (**C**) GO cellular component clustering heatmap. (**D**) GO molecular function clustering heat map. (**E**) KEGG biological pathway clustering heatmap. (**F**) Heatmap of the 13 SE-generated DEGs, with each column representing a sample and each row representing a gene. Color intensity indicates gene expression levels, with green indicating high expression and brown indicating low expression.

To identify key SE-regulated genes governing bovine myogenic differentiation, we integrated RNA-seq and ChIP-seq datasets from bovine MuSCs during GM and DM phases. SE annotation identified 3506 putative SE-associated genes, with 126 preferentially enriched in GM (Table S1) and 3380 in DM (Table S2, Figure 1A). Differential expression analysis revealed 17 SE-regulated genes significantly upregulated in GM compared to 377 in DM (Figure 1A). GO and KEGG analyses of SE-regulated genes during myogenic differentiation revealed significant enrichment in tissue development and muscle differentiation (biological processes), sarcomere organization (cellular components), and calcium ion binding (molecular functions) (Figure 1B–D). KEGG pathway analysis further demonstrated involvement in fluid shear stress/atherosclerosis, cell cycle regulation, and ECM–receptor interactions (Figure 1E). We randomly selected 13 SE-regulated genes focused on the myogenic differentiation period of bovine MuSCs to make the heat map (Figure 1F). In conclusion, these preliminary findings suggest the potential utility of SE-mediated transcriptional regulation in skeletal muscle development.

### 3.2. THBS3 Screening and Its Expression Distribution Characteristics

**Figure 2:**
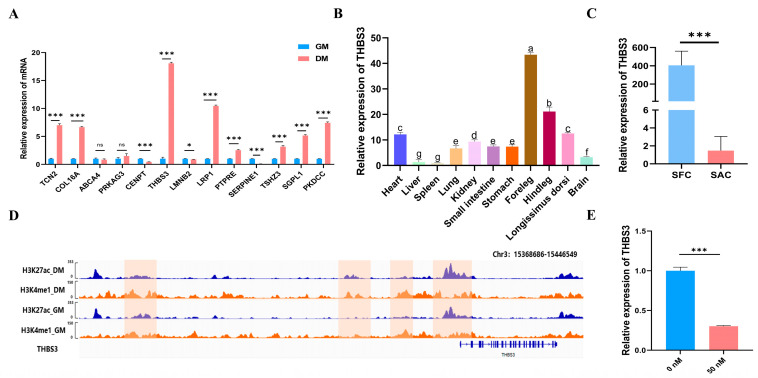
*THBS3* screening and its expression distribution characteristics. (**A**) Expression of differential genes at different stages (GM and DM). (**B**) Expression trend of *THBS3* in the longissimus dorsi muscle of bovine at different stages. (Fetus: SFC; Adult: SAC) (**C**) Expression levels of *THBS3* in different tissues during the embryonic stage of bovine. (**D**) Examples of ChIP-seq peaks marked with H3K4me1 and H3K27ac in GM and DM. The left side shows the cell stages and histone marks of the characterized MuSCs. The bottom shows the genes associated with the peaks. (**E**) The mRNA expression of *THBS3* after treatment with the JQ1 inhibitor. *ns* (not significant, *p* > 0.05), * *p* < 0.05, *** *p* < 0.001. Different letters above the bars denote significant differences among groups (*p* < 0.05). The results were presented as mean ± SEM of three replicates for each group.

RT-qPCR expression trends of 13 genes were highly consistent with the RNA-seq data (Figure 2A). Furthermore, we focused on the *THBS3* gene and found that, compared to the adult stage, *THBS3* was primarily enriched in fetal bovine muscle (Figure 2B) and exhibited distinct muscle-specific expression characteristics. In different fetal bovine tissues, *THBS3* was highly expressed in muscle-related tissues such as the foreleg and hindleg (Figure 2C). We further found that the SE activity at the *THBS3* locus significantly increased during myogenic differentiation (H3K27ac signal: GM 40 → DM 108, *p* < 0.001) (Figure 2D), and named this SE, *THBS3*-SE. To investigate whether *THBS3* was regulated by its SE, we treated bovine MuSCs with the BET inhibitor JQ1, resulting in a significant decrease in *THBS3* expression levels (Figure 2E), indicating that *THBS3* was regulated by its SE. In summary, these results suggested that the identified *THBS3* may play an important regulatory role in muscle development.

### 3.3. Interfering with THBS3 Promotes Proliferation of Bovine MuSCs

**Figure 3:**
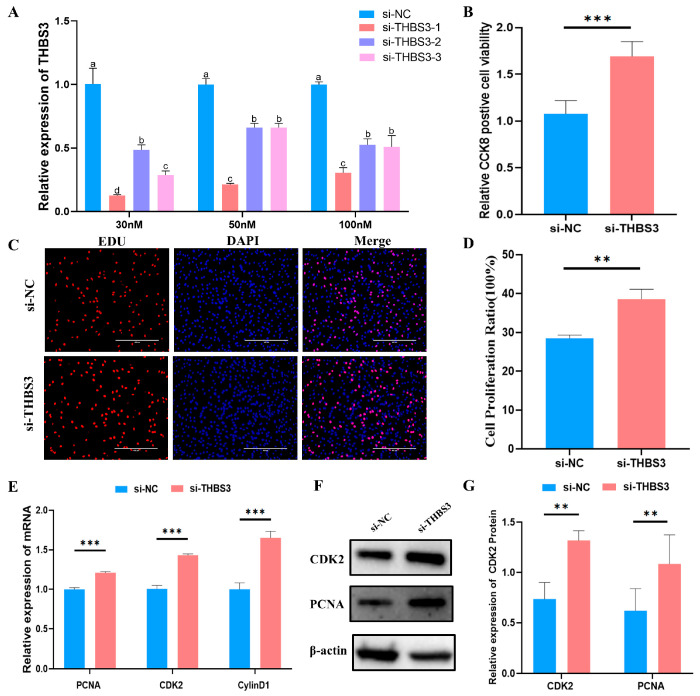
Interfering with *THBS3* promotes proliferation of bovine MuSCs. (**A**) RT-qPCR detection of the interference efficiency of different si-*THBS3* 24 h after transfection. (**B**) CCK8 detection of cell activity after interfering with *THBS3*. (**C**,**D**) Edu assessment of cell proliferation. (**E**) RT-qPCR analysis of proliferation markers (*PCNA*, *CDK2*) and cell cycle regulator (*CylinD1*) mRNA expression following *THBS3* knockdown. (**F**,**G**) Western blot analysis of CDK2 protein expression and PCNA protein expression levels after *THBS3* interference. ** *p* < 0.01, *** *p* < 0.001. Different letters above the bars denote significant differences among groups (*p* < 0.05). The results were presented as mean ± SEM of three replicates for each group.

To determine the role of *THBS3* in bovine MuSCs proliferation, we employed RNA interference (RNAi). First, we designed three siRNAs and transfected them into bovine MuSCs. RT-qPCR results showed that si-*THBS3*-1 exhibited the highest efficiency in inhibiting *THBS3* expression, achieving approximately 90% inhibition at a concentration of 30 nM (Figure 3A). Subsequently, CCK8 and EdU assays revealed that after *THBS3* expression was interfered with in MuSCs, cell viability significantly increased (*p* < 0.01, Figure 3B), and the number of EdU-positive cells significantly increased (*p* < 0.05, Figure 3C,D). Furthermore, RT-qPCR and WB analysis showed that interfering with *THBS3* not only significantly increased the mRNA expression levels of proliferation markers *PCNA*, *CDK2*, and *CyclinD1* (*p* < 0.01, Figure 3E) but also significantly increased the protein expression levels of CDK2 and PCNA (*p* < 0.01, Figure 3F,G). In summary, these results indicate that interfering with *THBS3* expression promotes the proliferation process of bovine MuSCs.

### 3.4. Interfering with THBS3 Promotes Myogenic Differentiation of Bovine MuSCs

**Figure 4:**
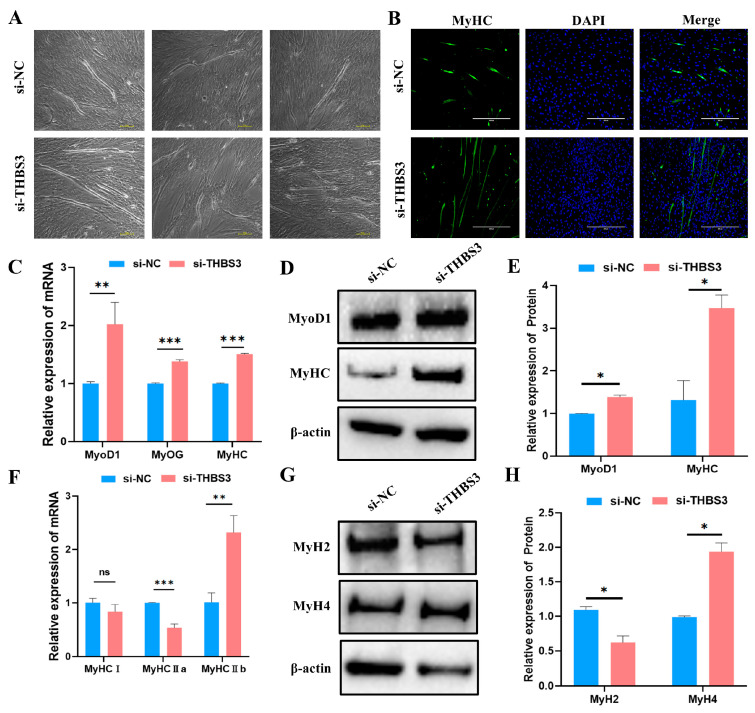
Interfering with *THBS3* promotes myogenic differentiation of bovine MuSCs. (**A**) After transfection with si-*THBS3*, induced differentiation of bovine MuSCs for 4 days resulted in the formation of numerous myotubes. Each group has three distinct fields of view. Scale bar: 100 μm. (**B**) Immunofluorescence analysis of the effect of *THBS3* interference on bovine MuSC myogenic differentiation. Scale bar: 400 μm. (**C**) RT-qPCR analysis of the expression of skeletal muscle differentiation marker genes (*MyoD1*, *MyOG*, *MyHC*) after transfection with si-*THBS3*. (**D**,**E**) WB analysis of MyoD1 and MyHC protein expression. (**F**) RT-qPCR detection of expression of myofibril-related marker genes. (**G**,**H**) WB analysis of protein expression of MyH2 and MyH4. *ns* (not significant, *p* > 0.05), * *p* < 0.05, ** *p* < 0.01, and *** *p* < 0.001. The results were presented as mean ± SEM of three replicates for each group.

To investigate *THBS3*’s function in myogenic differentiation, we quantified myotube formation efficiency and myogenic marker expression after RNAi-mediated *THBS3* knockdown in bovine MuSCs. Upon reaching 70–80% confluency, si-*THBS3*-transfected cells underwent differentiation for 4 days. Light microscopy images revealed enhanced myotube formation in si-*THBS3*-transfected cells (lower panel) compared to the si-NC control (upper panel), as observed in three fields of view (Figure 4A), validated by *MyHC* immunofluorescence (Figure 4B). The si-*THBS3* group consistently showed a higher density of multinucleated myotubes. Consistently, mRNA and protein levels of myogenic markers (*MyoD1*, *MyOG*, *MyHC*) were significantly upregulated (*p* < 0.05, Figure 4C–E). We further examined *THBS3’s* impact on fiber-type specification. RT-qPCR and WB showed significant downregulation of type IIa (fast oxidative) fiber marker *MyH2* (*p* < 0.05, Figure 4F–H). Concomitant upregulation of type IIb (fast glycolytic) fiber marker *MyH4* (*p* < 0.05, Figure 4F–H).

### 3.5. Screening of Differentially Expressed Genes and Functional Analysis After Interfering with THBS3

**Figure 5:**
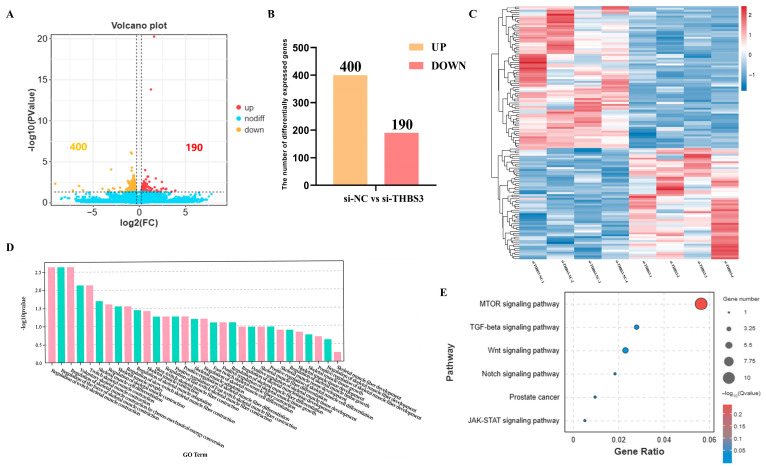
Screening of differentially expressed genes and functional analysis after interfering with *THBS3*. (**A**) Volcano plot showed the status of different genes in the two groups. Different colors representing differentially upregulated and downregulated genes screened according to the threshold, with black dots indicating no difference. (**B**) Statistical analysis of DEGs. Yellow indicates upregulated DEGs, and red indicates downregulated DEGs. (**C**) Hierarchical clustering analysis of DEGs. Each column represents a sample, and each row represents a gene. Gene expression was normalized using a z-score for each row. The color scale indicates the degree of gene expression, with red representing higher expression and blue representing lower expression. (**D**) Histogram of GO enrichment categories, with a significance threshold of *p* < 0.05. The *x*-axis represents secondary GO terms, while the *y*-axis represents the number of differentially expressed genes in each term. The color scheme indicates the direction of gene expression, with red indicating upregulation and green indicating downregulation. (**E**) Creation of a bubble chart for KEGG enrichment signaling pathways with a significance level of *p* < 0.05. This chart displays the gene count and enrichment level for each pathway, with bubble size representing the degree of enrichment and color intensity indicating the significance of the results.

We used transcriptomics to identify differentially expressed genes in the si-NC and si-*THBS3* groups, aiming to explore the regulatory mechanisms of myogenic differentiation of bovine MuSCs after *THBS3* interference. A total of 590 differentially expressed genes were identified between the si-NC and si-*THBS3* groups, including 400 upregulated genes and 190 downregulated genes (Figure 5A,B). Using GO annotation, the DEGs were classified into cellular components, molecular functions, and biological processes. GO terms were significantly enriched in skeletal muscle development-related pathways, including skeletal muscle contraction, skeletal muscle cell differentiation, and skeletal muscle fiber development (Figure 5D). KEGG pathway enrichment analysis showed that DEGs were primarily enriched in six pathways, including mTOR, TGF-β, Wnt, Notch, Prostate, and JAK-STAT signaling pathway (Figure 5E). Overall, these results indicated that *THBS3* was involved in muscle formation.

### 3.6. Identification of Chromatin Loop Structures of THBS3-SE and THBS3 Promoters

**Figure 6:**
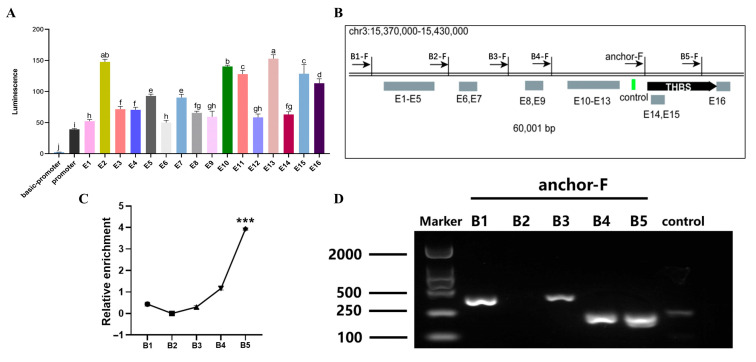
Identification of chromatin loop structures between *THBS3*-SE and *THBS3* promoter. (**A**) Detection of the activity of all splicing enhancers constituting *THBS3*-SE. (**B**) Specific sites for 3C detection were set for different predicted regions. (**C**) 3C-qPCR detection of the interaction frequency between enhancers and promoters at different sites. (**D**) Agarose gel electrophoresis validation of enhancer-*THBS3* promoter interactions. *** *p* < 0.001. Different letters above the bars denote significant differences among groups (*p* < 0.05). Results were presented as mean ± SEM of three replicates per group.

*THBS3*-SE was comprised of 16 constituent enhancers. Dual-luciferase assays in 293T cells identified six enhancers (E2, E10, E11, E13, E15, E16) with significantly higher transcriptional activity (*p* < 0.01, Figure 6A). Specific primer sets were designed across the locus (Figure 6B). 3C-qPCR analysis showed that high-frequency chromatin loop anchoring existed between the E15 and E16 regions (primer set B5-F) and between the *THBS3* promoter (*p* < 0.001, Figure 6C,D). These data demonstrated that E15 and E16 served as the functional core of *THBS3*-SE, physically engaging the *THBS3* promoter through chromatin looping to drive gene expression.

### 3.7. Inhibition of E15 or E16 Activity Promoted Proliferation and Myogenic Differentiation of Bovine MuSCs

**Figure 7:**
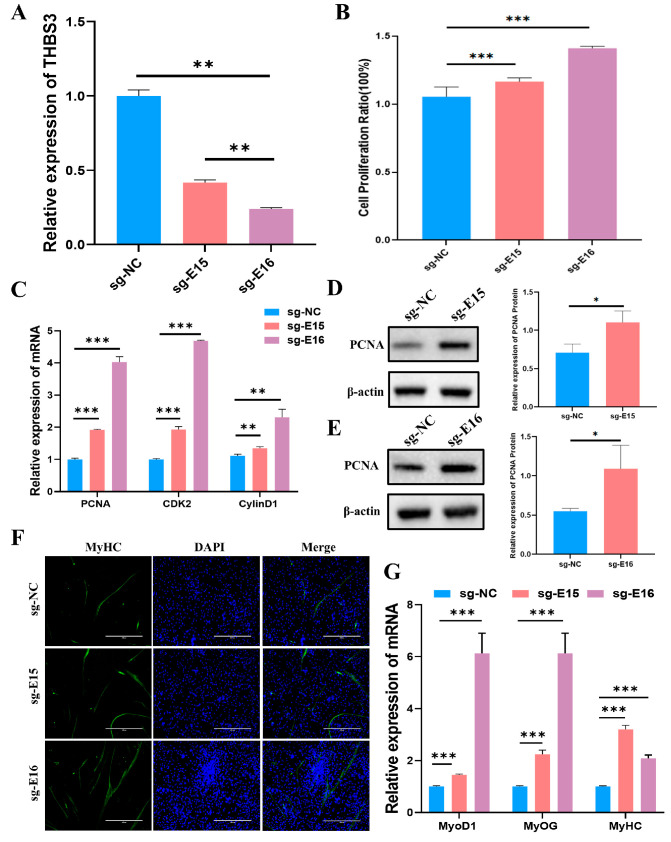
Inhibition of enhancers E15 and E16 activity promoted proliferation and differentiation of bovine MuSCs. (**A**) Expression levels of *THBS3* after inhibition of enhancers E15 and E16 activity. (**B**) Cell viability was assessed by CCK8 assay after inhibition of enhancers E15 and E16 activity. (**C**) RT-qPCR analysis of mRNA abundance of cell proliferation-related genes *PCNA*, *CDK2*, and *CyclinD1* after inhibition of enhancers E15 and E16 activity. (**D**,**E**) Analysis of PCNA protein concentration via WB. (**F**) Immunofluorescence staining after 4 days of induced differentiation following inhibition of enhancers E15 and E16 activity. *MyHC* (green) fluorescence indicates myotube formation, DAPI marks cell nuclei, and Merge shows the fusion between cell nuclei and myotubes. Scale bar: 400 μm. (**G**) After inhibiting enhancers E15 and E16 activity, induced differentiation for 4 days, and detected the mRNA levels of *MyoD1*, *MyOG*, and *MyHC*. * *p* < 0.05, ** *p* < 0.01, and *** *p* < 0.001. The results were presented as mean ± SEM of three replicates for each group.

To define the functional role of *THBS3*-SE in bovine myogenesis, we performed CRISPR interference (CRISPRi) by designing guide RNAs targeting enhancer E15 and E16. Validated sgRNAs guided dCas9-mediated repression (Supplementary Figure S2). This intervention significantly reduced *THBS3* mRNA levels (*p* < 0.01, Figure 7A). Subsequently, we assessed the effects on bovine MuSCs proliferation using CCK8, RT-qPCR, and WB. CCK8 assays revealed that inhibiting enhancers E15 and E16 activity significantly enhanced cell viability (*p* < 0.05, Figure 7B). RT-qPCR and WB detection revealed that the mRNA expression levels of *PCNA*, *CDK2*, and *CylinD1* were significantly increased (*p* < 0.01) (Figure 7C), and the protein levels of *PCNA* were significantly increased (*p* < 0.05) (Figure 7D,E). To investigate the effect of enhancers E15 and E16 on myogenic differentiation of bovine MuSCs, we used immunofluorescence staining to label *MyHC* protein in myotubes (Figure 7F). We observed that inhibiting the activity of enhancers E15 and E16 promoted myofiber fusion and increased myotube length. RT-qPCR analysis indicated that inhibiting the activity of enhancers E15 and E16 significantly promoted the expression levels of myogenic markers *MyoD1*, *MyOG*, and *MyHC* mRNA (*p* < 0.01, Figure 7G). In summary, CRISPRi-mediated repression of enhancers E15 and E16 within the *THBS3*-SE significantly enhanced both proliferation and myogenic differentiation capacity in bovine MuSCs.

## 4. Discussion

In mammals, prenatal myofiber formation is critically important, fundamentally determining the postnatal upper limit of total muscle fiber number and establishing the structural basis for postnatal hypertrophy [24]. Recent advances have identified novel regulators of bovine myogenesis development [21,25]. Here, we integrated and analyzed multi-omics data to identify *THBS3* as a key SE-regulated gene. It not only exhibited high expression during the DM phase but also displayed an elevated H3K27ac signal upstream of its genomic locus, a hallmark of active SE activity [26]. JQ1 inhibition experiment confirmed the presence of *THBS3*-SE. Furthermore, we examined the tissue-specific expression pattern of *THBS3* to elucidate its molecular function in organogenesis [27], which was significantly enriched in muscle tissue during the fetal stage of the bovine. Based on these findings, we inferred that *THBS3* was a key functional gene regulating skeletal muscle development in bovine.

Given its potential regulatory role, we employed RNA interference to define *THBS3*’s function in bovine MuSCs. *THBS3* knockdown significantly enhanced MuSCs proliferation, accompanied by upregulation of proliferative markers (*PCNA*, *CDK2*, *CylinD1*) at mRNA and protein levels. Interestingly, the proliferative effect observed upon *THBS3* silencing in bovine MuSCs directly contrasts with its reported role as a tumor suppressor in clear cell renal cell carcinoma (ccRCC) [28]. This functional divergence underscores profound cell-type-specificity and suggests that *THBS3* may operate within distinct molecular contexts. During the DM phase, *THBS3* interference promoted myotube formation, upregulated myogenic markers (*MyoD1*, *MyOG*, *MyHC*), and shifted fiber-type commitment from oxidative (Type IIa) toward glycolytic (Type IIb), suggesting its role in modulating meat quality. This biological function of simultaneously promoting MuSCs proliferation and differentiation following knockdown is similar to that of *MSTN* [29,30] and *FOXO1* [31,32]. Both processes are typically considered mutually exclusive [33], we found that both processes were enhanced by *THBS3* depletion, suggesting that *THBS3* may function as a critical regulatory node fine-tuning the transition from growth to differentiation phases in bovine MuSCs. In summary, *THBS3* emerged as a multifaceted regulator of bovine myogenesis.

To unravel the transcriptional networks through which *THBS3* influences myogenesis, we performed RNA-seq following *THBS3* knockdown. This revealed DEGs significantly enriched in biological processes critical to muscle development, such as skeletal muscle cell differentiation, regulation of muscle tissue growth, and myofiber development. *THBS3* is an adhesion glycoprotein that functions primarily in mediating cell extracellular matrix (ECM) interactions [34]. ECM regulates cell adhesion, migration, and differentiation through interactions with cell surface receptors, thereby controlling myofibrillar formation [35]. Based on this function, we speculated that it was a crucial component in establishing the cytoskeletal framework of MuSCs. *THBS3*’s potential mechanism of action may be similar to its role in cardiomyocytes [15], where it maintains the stability of the sarcolemma by moderately reducing integrin activity, thereby providing the necessary microenvironment for the normal differentiation and development of MuSCs. On the other hand, KEGG analysis further identified significant enrichment of DEGs in the mTOR signaling pathway. The mTOR signaling pathway orchestrates fundamental biological processes, including well-documented regulatory roles in sarcopenia [36,37] and skeletal muscle aging [38]. *THBS3* activated PI3K/AKT/mTOR signaling through protein kinase B (PKB) in gastric cancer [39]. Additionally, *THBS3* is enriched in ECM interactions and the PI3K-AKT-mTOR signaling pathway, thereby participating in the regulation of intramuscular fat in pigs [40]. Although our study is the first to demonstrate a robust link between *THBS3* knockdown and mTOR pathway dysregulation in Bovine MuSCs, the precise mechanism remains an open question. Notwithstanding these unknowns, our work established that *THBS3* may become a high-value target for bovine breeding. Its dual association with ECM integrity and mTOR signaling positions it as a unique regulator at the nexus of mechanical and biochemical cues governed by myogenesis.

SEs exhibit vastly superior transcriptional regulatory capacity compared to typical enhancers, attributable to their efficient recruitment of high-density transcriptional complexes [13,41]. Most SE are associated with cell lineage-specific identities. In myogenesis, established SEs such as *FoxO*-SE [42], *MyoD1*-SE [43,44], and *CASZ1*-SE [45] critically regulate muscle differentiation. Here, we delineated *THBS3*-SE as a conserved SE that orchestrates bovine MuSCs’ fate decisions by coordinately regulating proliferation and differentiation. Comprising 16 constituent enhancers, *THBS3*-SE harbored six high-activity elements (E2, E10, E11, E13, E15, E16). SEs commonly achieve target gene activation through long-range chromatin looping, exemplified by *MYC* regulation in epithelial cancers via SE-driven chromatin architecture [46] and SUCLG2-AS1 transcript-mediated *SOX2* control in nasopharyngeal carcinoma [47]. Enhancers E15 and E16 exhibited significantly higher interaction frequencies with the *THBS3* promoter compared to other enhancers, the finding confirmed by 3C-qPCR. Thus, we demonstrated that core enhancers E15/E16 formed chromatin loops with the *THBS3* promoter to directly regulate transcription. However, SEs may coordinate multiple genes via looping and recruit TFs to target promoters [13,48]. Beyond *THBS3*, the full regulatory network of *THBS3*-SE warrants future elucidation using HiChIP-seq assays [49,50]. The functional roles of most SEs have been characterized in cancer contexts [47,51,52], revealing promising therapeutic targets. Our study in bovine MuSCs demonstrated that inhibiting enhancer E15 and E16 activity significantly reduced *THBS3* mRNA levels, an effect comparable to BET inhibitor JQ1 treatment, confirming SE-dependent regulation specificity. Contrastingly, Cui et al. reported that deletion of any constituent enhancer within SEs downregulated *EphA2* in multiple cancer cell lines (HeLa/HCT-116/MCF-7) [53], potentially attributable to conserved chromatin architecture. Although this study focused on enhancer E15/E16 due to their maximal interaction with *THBS3*, we still did not know whether other enhancers participate in the regulatory process. Notably, inhibition of enhancer E15 and E16 activity similarly significantly enhanced both proliferative capacity and myogenic differentiation efficiency in bovine MuSCs. This phenotypic parallelism functionally recapitulated the outcomes of direct *THBS3* perturbation in bovine MuSCs. Collectively, enhancer elements E15 and E16 constituted the core architectural units of *THBS3*-SE, orchestrating target gene transcription via chromatin looping with the *THBS3* promoter, ultimately governing MuSCs proliferation and myogenic differentiation potential.

## 5. Conclusions

This study established a novel epigenetic mechanism wherein the *THBS3*-SE coordinates bovine muscle stem cell proliferation and differentiation via a specific chromatin loop formed between its core enhancers (E15/E16) and the *THBS3* promoter. Functionally, we identified both *THBS3*-SE and its target gene *THBS3* as key negative regulators that restrain myogenic progression. Central to this regulatory axis is the E15/E16-mediated chromatin looping, which directly drives *THBS3* expression and sustains its repressive influence on muscle development. These findings not only revealed a previously unidentified signaling pathway in bovine myogenesis but also highlighted *THBS3*-SE and *THBS3* as attractive targets for precision breeding strategies. By modulating these elements, it may be possible to enhance muscle growth and meat quality in cattle. Our work thus provided a genetic and epigenetic framework for future innovations in beef production through targeted regulation of skeletal muscle development.

## Data Availability

No data were deposited in an official repository. All data generated during this study are available from the corresponding author upon request.

## Supplementary Materials

The following supporting information can be downloaded at https://www.mdpi.com/article/10.3390/ani15172615/s1. Table S1a: SE-associated DEGs during GM. Table S2a: SE-associated DEGs during DM. Supplementary Table S1b: Gene-specific primer pairs used for RT-qPCR and Vector Construction. Supplementary Table S2b: siRNA, Vector, and 3C-Specific primer Construction. Supplementary Figure S1: Variations in phenotypic characteristics and markers during proliferation and differentiation of bovine muscle stem cells. Supplementary Figure S2: Agarose gel electrophoresis and Sanger sequencing confirmed the construction of active inhibition vectors. The material information about E15–E16 motif can be uploaded from Supplementary Files.
