# Supplementary material for: Super-Enhancer Drives THBS3 Expression to Regulate the Proliferation and Differentiation of Bovine Muscle Stem Cells
Source: Animals (Basel). 2025 Sep 6;15(17):2615. doi: 10.3390/ani15172615 (PMC12427415; doi:10.3390/ani15172615)
Supplement: Supplementary file 1 [file animals-15-02615-s001.zip › Supplementary Materials.pdf]

Supplementary Materials

Supplementary Table S1b. Gene-specific primer pairs used for RT-qPCR and Vector Construction.

Genes	Sense primer (5'→3')	Antisense primer (5'→3')	Product length (bp)
THBS3	ACTTTGAGCCATTCCGGAGG	CTCCTGAACAGTGGGGTCAC	130
TCN2	TGCCTCCAACAAGGATGACA	CACACTGTCGTGGACTCGCTT	265
COL16A	AGGGACCATTCATCTTGCGA	TGCTCCTGACTGTTGACTTCCA	205
ABCA4	GGGTGTTCTCCTTCAACTGGTAT	TGCAGGGGAATCTGGAGTAAA	221
PRKAG3	TCCTCTACCGCACCATCCAA	AATCACATCAAAGCGGGAGTAGA	179
CENPT	TAGGAGGCAGAGGAGTCAAACAA	TTGTGGGGGGCTCAAGTTC	281
LMNB2	TGTCTTTGAGGAGGAAGTGCGT	AGCCTTGTCATTCTGGTCGGA	226
LRP1	TCTACTGGGCTGATGCCTACCT	TACAGGTGCTCAATCAGGATGC	100
PTPRE	GGTCAGTGCCAACGACAAGAA	ATTGGGAAGGATGTTGGGG	280
SERPINE1	CAACTTCTTCAGGCTGTTCCGT	TTGACTCTGGGAAGGGCATC	215
TSHZ3	CATCCCACCAGAAGACACGAC	GACCAGTAGGAGTTGGAGAGGAAG	87
SGPL1	GACCAGTAGGAGTTGGAGAGGAAG	TGTATGAGTTTGTCTTCCAGCCA	163
PKDCC	AACGCAACCTCTACAATGCCTAC	CAGGTTGAACACGGAAAGGAG	311
PCNA	GAACCTCACCAGCATGTCCA	ACGTGTCCGCGTTATCTTCA	86
CDK2	TCTTTGCTGAGATGGTGACCC	TAACTCCTGGCCAAACCACC	117
CylinD1	TGGAGCCCGTGAAAAAGAGC	CCGGATGGAGTTGTCAGTGT	1128
MyOD1	CAGCAAGTTTCTGGCAACCC	AAGTTGCAGAGAGAGCCGTC	144
MyOG	GCGCAGACTCAAGAAGGTGA	TGCAGGCGCTCTATGTACTG	126

MyHC	TGCTCATCTCACCAAGTTCC	CACTCTTCACTCTCATGGACC	150
MyHC I	AGCAGCCTCTTTGCCAACTAT	CAGAGCTGACACGGTCTGAA	99
MyHC IIa	GGACCAAGTGAACGAGCTGA	GCCCCTTGACAACTGAGACA	154
MyHC IIb	GGCACCGTGGACTACAACAT	CACTTGGACCTCCAGCGAAG	130
β-actin	CATCCTGACCCTCAAGTA	CTCGTTGTAGAAGGTGTG	91
pGL3-E1	CGTGCTAGCCCGGGCTCGAGGGGACTAGATGACGCTTAAGGT	TGCAGATCGCAGATCTCGAGGCCCATGCTCTGAGCTCC	444
pGL3-E2	CGTGCTAGCCCGGGCTCGAGAGGGGTACAGGACACCCT	TGCAGATCGCAGATCTCGAGCAGCCACTGTAAGGAGGAGAAA	374
pGL3-E3	CGTGCTAGCCCGGGCTCGAGCCTCCGTGAGGCCTCC	TGCAGATCGCAGATCTCGAGCCAGCCCAAGACGGGCA	408
pGL3-E4	CGTGCTAGCCCGGGCTCGAGGCGTTTTCCGGGGTTGTTG	TGCAGATCGCAGATCTCGAGGCAATTTTCAGTTCCTGCCCC	548
pGL3-E5	CGTGCTAGCCCGGGCTCGAGTCTATTAGGCTTTGGCATGGACA	TGCAGATCGCAGATCTCGAGCAAAACAAAGGGGTAGGGAGTG	593
pGL3-E6	CGTGCTAGCCCGGGCTCGAGGTTAGATAAGTGCTGTGTGCTTAGT	TGCAGATCGCAGATCTCGAGATTTCTTATGTTGCATGGGCTGAAG	321
pGL3-E7	CGTGCTAGCCCGGGCTCGAGGCCGTGCAAAGTTGCTTATGC	TGCAGATCGCAGATCTCGAGCTCTGGGGCAGAGTAACTG	293
pGL3-E8	CGTGCTAGCCCGGGCTCGAGCTCTGTCCTGTGAGAAAGGAAAAT	TGCAGATCGCAGATCTCGAGCAGCTTTAGGAAGAATCTAGATGAA	456
pGL3-E9	CGTGCTAGCCCGGGCTCGAGTCCCGCCGTGGGCTTCAT	TGCAGATCGCAGATCTCGAGGCCAACTCTTCTCTGACCC	1289
pGL3-E10	TGCAGATCGCAGATCTCGAGGCCAACTCTTCTCTGACCC	CGTGCTAGCCCGGGCTCGAGCCGCTGCATGCGGCCA	486
pGL3-E11	TGCAGATCGCAGATCTCGAGACTGGGCCCAGGCGAG	CGTGCTAGCCCGGGCTCGAGAACTTCCCCAATCCAGACACCA	413
pGL3-E12	TGCAGATCGCAGATCTCGAGTTCTCCTACCACAGCCATATTGA	CGTGCTAGCCCGGGCTCGAGCACATAGCCACAACACACCCTT	506

pGL3-E13	TGCAGATCGCAGATCTCG AGGGTAAGCCTGTACCAA TCCTCT	TGCAGATCGCAGATCTCGA GTCCTGTGTCCTGGCCAAT CT	504
pGL3-E14	TGCAGATCGCAGATCTCG AGTCCTGTGTCCTGGCCA ATCT	CGTGCTAGCCCGGGCTCGA GTGCCTTGTCCTCTGATAGC AAC	312
pGL3-E15	CGTGCTAGCCCGGGCTCG AGGACCCTCGGGCCTTGG	TGCAGATCGCAGATCTCGA GAGAGCGGGATTCCGGGC	596
pGL3-E16	CGTGCTAGCCCGGGCTCG AGCCACCACCTTGTGCGT AC	TGCAGATCGCAGATCTCGA GATGGAGGGATTGGGGGTT TTAG	269

Supplementary Table S2b. siRNA , Vector and 3C- Specific primer Construction.

Name	Primer sequence 5'→3'
si-*THBS3*-1	CGTCGGCAAGATCAACAAA
si-*THBS3*-2	TCCGAAACACCATCATGGA
si-*THBS3*-3	CTGTGACAACGACGTAGAT
Px330a dCas9-E15-1	CACCGAGTGTCGCCGCTGCCACCGT
Px330a dCas9-E15-2	CACCGCCCGGCTGCTCTTAGACTG
Px330a dCas9-E16-1	CACCGATTTGGGCGAGGACATCGGA
Px330a dCas9-E16-2	CACCGTTTGGGGCAGGTACGCACA
Anchor-F	CGAGCAGCAAACTTCATTCC
Control	F: CTACTCCTGTCCTTGGTATC<br>R: GGAATGAAGTTTGCTGCTCG
3C-B1	F: GTAGAGCAGAAAGCAGCCAAG<br>R: GTCCACCTTACCTTCTCTAG
3C-B2	F: CATCTAGTTGCTGGCAGACA<br>R: CTAACCTGTATCTGCAGAGG
3C-B3	F: CTTCAGTTGGGCTTCCCTTG<br>R: CCTCAATCGTGTCCAACTG
3C-B4	F: CTGGTGATGGAGCTTTCAAG<br>R: CACTTCTCCTTCAATCCCAC
3C-B5	F: AGGTATAGGGGAATGGAGAG<br>R: CACTAGGTCACTGTCTGCATC

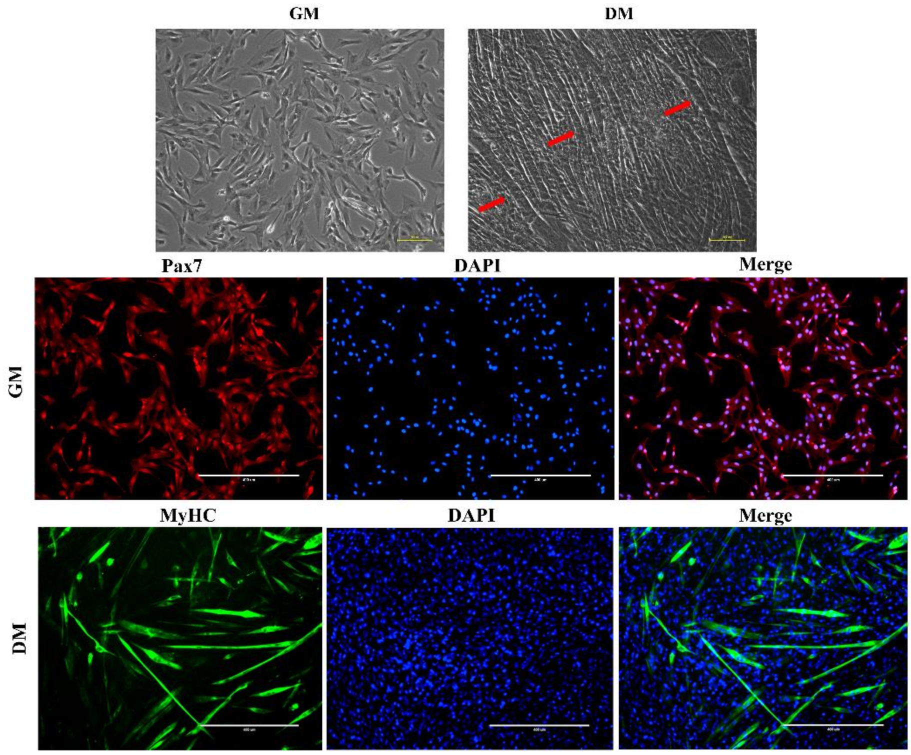

Figure S1. Variations of phenotypic characteristics and markers during proliferation and differentiation of boivne muscle stem cells

After passage culture, bovine MuSCs exhibited good growth status during the GM and formed three-dimensional and robust myotubes after induction of DM. These cells expressed the MuSC-specific marker protein Pax7, and the myotubes formed after induction of differentiation were enriched with the myogenic marker molecule MyHC.

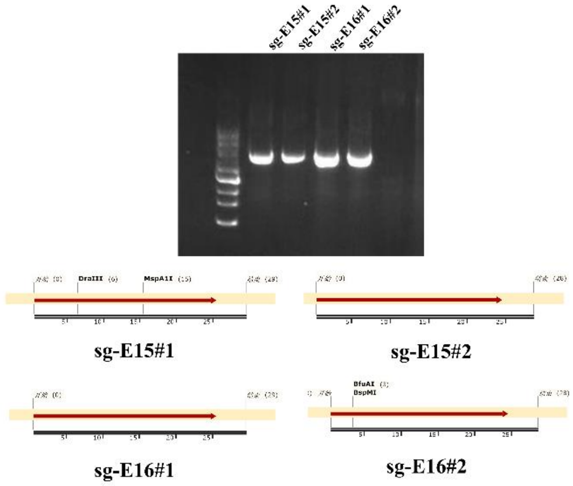

Figure S2. Variations of phenotypic characteristics and markers during proliferation and differentiation of boivne muscle stem cells

Agarose gel electrophoresis showed successful construction of active inhibition vectors E15 and E16. Sanger sequencing confirmed the sequence fidelity of active inhibition vectors E15 and E16.
